# Commentary: Genetic evolution of classical swine fever virus under immune environments conditioned by genotype 1-based modified live virus vaccine

**DOI:** 10.3389/fvets.2018.00055

**Published:** 2018-03-21

**Authors:** Liliam Rios, Lester J. Pérez

**Affiliations:** ^1^Reiman Cancer Research Laboratory, Faculty of Medicine, University of New Brunswick, Saint John, NB, Canada; ^2^Dalhousie Medicine New Brunswick, Dalhousie University, Saint John, NB, Canada

**Keywords:** classical swine fever virus, positive selection pressure, neutralization-escape mutant, global evolutionary patterns, modified live virus vaccine

Classical swine fever (CSF) is a major pig disease worldwide (1). Some research studies have focused on developing new control policies, especially for CSF-endemic countries. A recent study by Yoo et al. (2) described the genetic evolution of CSF virus (CSFV) under immune environments conditioned by genotype 1-based modified live virus vaccine (MLV). Based on their results, the authors suggest there is a need to develop a new CSFV vaccine based on CSFV-genotype 2 (CSFV-G2) (2). However, as discussed below, the main findings of this study were not properly supported by the results or by the choice of experimental design.

Analyzing the global evolutionary patterns for CSFV, Yoo et al. (2) stated that the genetic diversity of the CSFV-G2 was higher than that of the CSFV-genotype 1 (CSFV-G1). In this experiment, the authors compared the effective population size (Ne) vs time between both genotypes (2). The authors suggest that the Ne values for the CSFV-G1 remained relatively constant whereas, for CSFV-G2, the Ne values gradually expanded after 1980 (2). However, by looking at the effective population size (*y*-axis), it is clear that the Ne for CSFV-G1 was higher (around 10^2–2.5^) compared to the Ne values of CSFV-G2 until the year 2000. Between 2000 and 2005, there is a sudden increase in the Ne for CSFV-G2 followed by an almost equal decrease. The Ne values remain higher for CSFV-G1 compared to CSFV-G2, plateauing at approximately 10^2.5^ after 2009 at the time when the genetic diversity of the CSFV-2 continues to decrease. Moreover, any comparison after this point is difficult to assess since the authors did not continue their analysis for CSFV-G1 after 2010, unlike that of CSFV-G2 (2). This raises an additional concern regarding the inconsistency of this study, since CSFV-G1 sequences collected after 2010 are available on GenBank databases and have been used in phylodynamic studies for CSFV-G1 (3–5). Finally, in Figure 3, the genetic diversity is expressed by the median estimate of the Ne (solid line) with a 95% highest posterior density (HPD) interval (gray area) (2). Considering the 95% HPD, there is no statistical difference between these two populations. From our analysis, the fact that the genetic diversity of CSFV-G1 showed higher values than CSFV-G2 consistently over a longer period of time (2) is an indication that CSFV-G1 has higher diversification than CSFV-G2, contradicting the conclusions made by the authors.

Second, the author’s state: “*CSFV-G2 has a more advantageous E2 codon composition than CSFV-G1, in terms of survival in immune environments that have been optimally created by CSFV genotype 1-based vaccination.”* This conclusion was not supported by the methods used. For the evaluation of the selective pressure on CSFV, Yoo et al. (2) employed the estimation of the ratio of non-synonymous to synonymous substitution rates using four different testing methods implemented in HYPhy package. However, these methods were designed to determine the action of the evolutionary forces on codon sites, but not to compare evolutionary advantages between lineages. Currently, the only program to evaluate evolutionary advantages between lineages is PAML, since branch-site models are implemented in this program (6). For this reason, the interpretation of the results by Yoo et al. (2) was not properly supported. Alternatively, Rios et al. (5) showed, using a branch-site model, that the only CSFV lineage selected by positive selection was subgenotype 1.4 (5). When using this same model at the genotype level, no evidence of evolutionary advantage by the action of positive selection pressure for any of the CSFV genotypes assessed was observed (5).

Because of the emergence of neutralization-escape mutants from the CSFV-G2 strains caused by the disproportionate use of MLV based on CSFV-G1, Yoo et al. (2) proposed that there is a need to develop a new CSFV vaccine based on CSFV-G2 to prevent vaccine-escaping mutants of this genotype. However, no experimental designs supporting this statement were included in this study. Yoo et al. (2) restricted their study to describe some amino acid substitutions found in the analyzed sequences. Based on these substitutions, the authors conclude these are neutralization-escape mutants. Experiments using monoclonal or polyclonal antibodies would have provided the necessary information to claim these mutants were indeed neutralization-escape mutants.

A series of studies (3, 7–9) previously demonstrated the emergence of a neutralization-escape mutant for CSFV strains from the subgenotype 1.4. In Perez et al. (3), the authors found that the vaccination policy implemented in Cuba (CSF-endemic) led to a bottleneck effect on the viral population in this country, causing the emergence of new strains. Further studies revealed that one of the strains suggested to be a neutralization-escape mutant showed lower virulence compared to the parental strain (7, 8) and induced postnatal persistent infection, representing an evolutionary advantage (8). Coronado et al. (9) compared the antigenic relationships between the parental strain that circulated in Cuba (“Margarita strain”) and the new emergent strain (“Pinar del Rio”), as well the capacity of neutralization induced by the MLV implemented in Cuba for both these CSFV strains (9). The results from these studies showed antigenic differences between the parental strain and the emergent strain when values of neutralization antibodies (homologous and heterologous) were compared. Furthermore, whereas the immune response induced by the MLV vaccine applied in Cuba was able to completely neutralize the parental strain “Margarita,” it was only able to partially reduce the emergent strain “Pinar del Rio” (9). This provides evidence that the MLV based on CSFV-G1 can induce neutralizing-escape mutants in the same genotype due to positive selection pressure. It is probable that the MLV based on CSFV-G1 1 could also induce the emergence of neutralizing-escape mutants in the CSFV-G2, however, the study published by Yoo et al. (2) did not show any evidence in this regard.

Relevant factors that could facilitate the emergence neutralization-escape mutants for CSFV were omitted in Yoo et al. (2). These include: the composition of the quasispecies cloud (10), the circulation of immunosuppressive (11), and the properties of the vaccine (quality, doses, gaps in the cold chain) (1). Therefore, the suggestion to produce a CSFV vaccine based on CSFV-G2 to avoid the emergence of neutralizing-escape mutants of this genotype lacks sufficient supporting evidence.

## Author Contributions

LR and LP wrote the manuscript. LP edited the manuscript. Both authors read and approved the final version of the manuscript.

## Conflict of Interest Statement

The authors declare that the research was conducted in the absence of any commercial or financial relationships that could be construed as a potential conflict of interest.
